# COVID-19 Presenting as a Seizure: A Kenyan Case Report

**DOI:** 10.7759/cureus.24431

**Published:** 2022-04-24

**Authors:** Syed Moosa Raza, Farah Ebrahim, Herman Ekea, Sayed K Ali

**Affiliations:** 1 Medicine, University of Central Florida, Orlando, USA; 2 Internal Medicine, The Aga Khan University Hospital, Nairobi, KEN; 3 Neurology, The Aga Khan University Hospital, Nairobi, KEN

**Keywords:** neuronal hyperexcitability, management, kenya, epileptic seizures, covid-19

## Abstract

The new coronavirus quickly spread throughout the world in late 2019 and became a pandemic in early 2020. The most common symptoms observed are fever, dry cough, loss of taste and smell, and respiratory distress. Other rarer complications can involve the cardiovascular, gastrointestinal, or neurological systems. Of the neurological complications, epileptic seizures are a subject of particular interest due to their relatively unknown and widespread etiologies. It is understood that the entry or production of pro-inflammatory cytokines during a COVID-19 infection can result in neurotransmitter modulation and ion channel dysfunction, leading to neuronal hyperexcitability, presenting as seizures. To the best of our knowledge, we present the first case in sub-Saharan Africa of a COVID-19 positive patient presenting to our institution with a reported seizure followed by confusion. Our case highlights the need to broaden our differential diagnosis to include COVID-19 infections in patients presenting with seizures.

## Introduction

The coronavirus disease 2019 (COVID-19), caused by the novel coronavirus (SARS-COV-2), has rapidly spread worldwide since late 2019. It continues to be a significant global health threat in many parts of the world, owing to new variants that have increased virulence or transmissibility and decreased the effectiveness of public health and social preventative measures. Major symptoms of infected patients include fever, dry cough, pain, body aches, chills, lethargy, anorexia, anosmia, and ageusia [1]. Some patients may also experience cardiovascular complications such as heart failure, coagulation disorders, irregular heart electrical activity, and gastrointestinal (GI) complications such as anorexia, nausea, diarrhea, vomiting, and abdominal pain [2]. In particular, a minority of the patients may present with neurological symptoms such as headaches, paresthesia, anosmia, ageusia, vertigo, delirium, ischemic/hemorrhagic stroke, encephalitis, and seizures [3,4]. While the exact mechanisms of seizures are not yet completely understood, the consensus is that they are caused by neuronal hyperexcitability following ion channel dysfunction. This could result from increased excitatory neurotransmitters such as aspartate or glutamate or the decrease of Gamma-aminobutyric acid (GABA), an inhibitory neurotransmitter [5]. Other likely causes include electrolyte imbalances, hypo or hyperglycemia, and acute neuronal damage resulting from infection, inflammation, head trauma, stroke, fever, and hypoxia [6]. To the best of our knowledge, we present the first observed case of a patient with a COVID-19-induced seizure in Kenya.

## Case presentation

A 33-year-old male patient with no other medical comorbidities presented to our institution with a 48-hour history of sudden on-set dizziness preceded by an episode of diaphoresis. Twenty-four hours into his symptoms, he had an episode of tonic colonic seizure, lasting approximately one minute, with urinary and stool incontinence. There were no episodes of tongue biting, eye-rolling, or frothing of the mouth. He appeared confused for approximately three minutes, as per the family, and after that felt a sensation of fatigue. The seizure episode was reported by his wife and partially recorded by a family member. 

On presentation to the emergency room, he was tachycardic at 123 beats per minute, and he had a regular respiration rate and normal temperature of 36.5 degrees centigrade. His neurological exam, in particular, was within normal limits. The rest of his physical examination was unremarkable. He denied any travel history or exposure to sick contacts.

**Figure 1:**
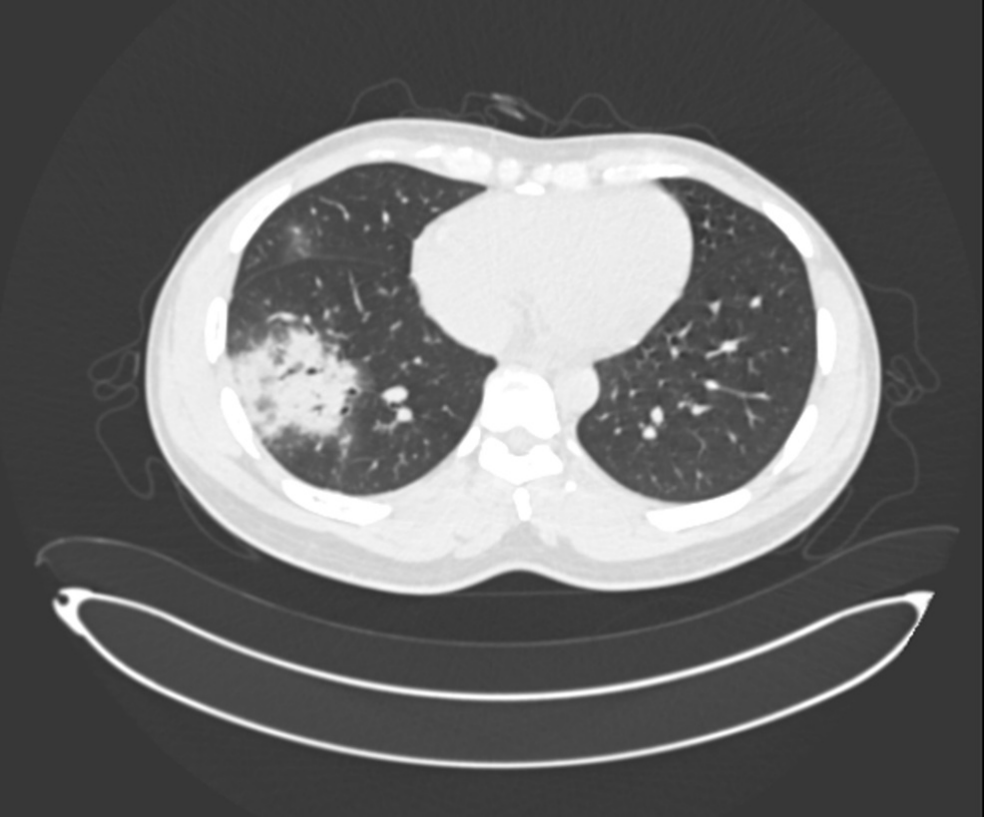
HRCT features of an atypical pneumonic with less than 10% parenchymal involvement.

His initial laboratory workup showed a glucose level of 133mg/dL, white cell count of 6.98 x10^3^μL (normal neutrophil count), hemoglobin of 14.5g/dL, and platelets 159μL. His extended electrolytes were also within normal limits. The creatinine was slightly elevated at 117 μmol/L with normal potassium of 3.82mmol/l, urea 4.4mmol/l, and sodium of 144mmol/l. The HBA1c was reported at 6.1%. His CRP was slightly elevated at 16mg/dL, and a procalcitonin was reported at 0.11ng/mL. His D-dimer was also slightly elevated at 2.0µg/ml. Urine toxicology was negative for any illicit drugs. His RT-PCR for COVID-19 was reported positive 24 hours after presentation. A high-resolution CT (HRCT) of his chest was suggestive of atypical pneumonia with 10 percent of lung involvement (Figure 1).

**Figure 2:**
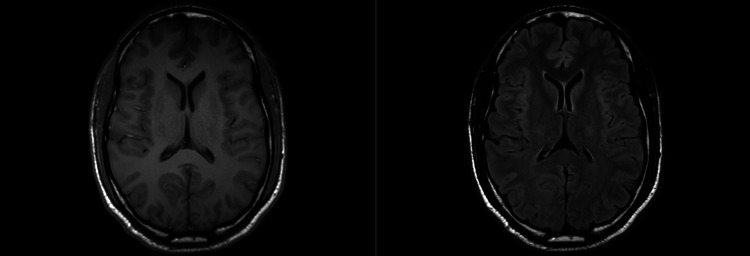
Unremarkable MRI of the brain

A subsequent lumbar puncture showed a white cell count and a red cell count of zero. The cerebrospinal fluid (CSF) protein and glucose levels were within normal limits, and cultures were reported as negative. CSF cultures for tuberculosis (TB) were also negative, and COVID-19 PCR of the CSF was not done. An electroencephalogram (EEG) was reported as normal, and a subsequent MRI of the brain was reported as unremarkable (Figure 2). A 2D echocardiogram showed a 55-60% normal ejection fraction with no wall abnormalities and normal valvular function.

He was initially loaded with Levetiracetam at 1gm and continued on a regular dose at 500mg twice daily. He also received prophylactic enoxaparin at 40mg subcutaneous once a day. The patient faired well until hospital day two of admission when he started spiking fevers of 38.5^o^C with a persistent tachycardia of 120 - 130 beats per minute. His oxygen saturation also dropped to less than 92%. He was immediately started on oral dexamethasone 6mg a day and supported with oxygen (2 liters) to maintain saturations of about 92%. Within 72 hours, he was off oxygen, and no additional seizures were reported during his hospital stay. He was discharged on hospital day six to complete 14 days of self-isolation at home.

## Discussion

SARS-COV-2 can penetrate the nervous system via the hematogenous route by affecting the endothelial cells of the blood-brain barrier or leukocytes, eventually causing dissemination to other tissues. Alternatively, the virus can also enter the nervous system through peripheral nerves retrograde via active axonal transport [7]. SAR-COV-2, like SARS and MERS, is also thought to bind to the Angiotensin-converting enzyme 2 (ACE2) receptors found on the meninges and blood-brain barrier, making the tissues susceptible to injury and eventually resulting in neuronal cell death [7,8]. The SAR-COV-2 virus can also directly enter the brain through the olfactory tract, surpassing the need for ACE2 receptors [8]. Once the virus has invaded the central nervous system (CNS), it triggers reactive astrogliosis and stimulates the microglia to induce a large inflammatory cascade, which results in a cytokine storm.

Pro-inflammatory cytokines such as tumor necrosis factor-alpha (TNF-α), Interleukin 1 beta (IL-1B), Interleukin 6 (IL-6), free radicals, nitric oxide, and others are released, which leads to acute or chronic inflammation, causing neuronal hyperexcitability and potentially resulting in seizures [1]. These cytokines aggravate apoptosis and neuronal necrosis in the CNS, and they also stimulate glutamate release while inhibiting GABA release in the hippocampus and cerebral cortex (IL-1B, TNF-α) [9]. Additionally, using α-amino-3-hydroxyl-5-methyl-4-isoxazole-propionate (AMPA) and N-methyl-D-aspartate (NMDA) receptors, these cytokines increase calcium entry into the neurons, leading to neuronal hyper-excitability (TNF-α). Lastly, they release various neurotoxic compounds through different autocrine/paracrine mechanisms (IL-6). Furthermore, IL-6, in particular, has been shown to contribute to a fever response. This specific pro-inflammatory cytokine can play a role in neuroinflammation and seems to contribute to the inflammatory signaling on the seizure threshold [10]. Some studies have also linked IL-6 to the development of seizures [11]. Since COVID-19 is associated with an elevated level of IL-6 and fevers, it could be hypothesized that this increases the possibility of developing seizures in certain patients.

Of note, there has been no definitive association between COVID-19 and seizures. However, fear of the infection and isolation resulting in stress and elevated levels of cytokines has been known to lower the seizure threshold and contribute to the development of seizures [12,13]. Lu et al. conducted a retrospective study looking at seizures among patients with COVID-19. They found that hypoxia, the most common complication in their cohort studied, could potentially trigger anoxic encephalopathy, causing seizures [13]. Hypoxia due to COVID-19 has also been shown to result in ischemic stroke, which can augment the development of seizures in certain patients [14]. In addition, electrolytes abnormalities and certain antibiotic use could potentially trigger seizures in patients [13]. 

In every COVID-19 patient presenting with seizures, it remains imperative to investigate the seizures' etiology further. This includes a careful review of their medications, a complete metabolic investigation, a prolonged EEG (if available), and brain imaging. CSF analysis is also necessary to further investigate acute viral causes of seizures [15]. Of note, many studies have reflected on their inability to detect the SARS-COV-2 virus in the CSF of COVID-19 patients [15].

Our patient was initially treated with the Anti-Epileptic drug (AED) Keppra (Levetiracetam). While the exact mechanism for Levetiracetam's anti-epileptic activity is not entirely understood, studies have shown that it prevents GABA inhibition. This presynaptic inhibitor suppresses glutamate release [16], and it exhibits inhibitory effects on calcium release, thereby inhibiting the release of Ca2+-associated neurotransmitters [17]. Unlike the other AEDs, Levetiracetam has the least number of interactions with other COVID-19 related therapies and is the preferred choice of treatment in patients with seizures and COVID-19 [18]. Usually, patients do not require prolonged anti-seizure therapy after the acute episode has resolved and in the absence of repeat seizures [15,19]

On follow-up in the clinic 2 and 6 weeks later, our patient did well with no reported seizures. His Levetiracetam was stopped, and he continues to do well currently. COVID-19 specific vaccinations, approximately 12 weeks post his initial infection, were recommended to help mitigate future infections.

## Conclusions

One of the rare neurological complications of COVID-19 is seizures, which can occur due to neuronal hyperexcitability induced by neurotransmitter or electrolyte modulation due to the cytokine storm-generated due to inflammation during the body's immune system response. A thorough workup is required better to understand the etiology of seizures in COVID-19 patients. Treatment with drugs that reverse the upregulation/downregulation of these neurotransmitters or electrolytes, thereby restoring proper ion channel function in the CNS, can ameliorate pathogenicity. 
